# Neuroendocrine small cell rectal cancer metastasizing to the liver: a unique treatment strategy, case report, and review of the literature

**DOI:** 10.1186/1477-7819-11-153

**Published:** 2013-07-11

**Authors:** Bilal O Al-Jiffry, Owaid Al-Malki

**Affiliations:** 1 Department of Surgery, College of Medicine and Medical Sciences, Taif University, P.O. Box 888, Taif 21947, Kingdom of Saudi Arabia; 2 Department of Surgery, Al-Hada Military Hospital, P.O. Box 1347, Taif, Kingdom of Saudi Arabia

**Keywords:** Extrapulmonary small cell cancer, Rectal cancer, Neuroendocrine liver metastasis, Large liver metastasis, Extended liver resection, Abdomino-perineal resection

## Abstract

We describe the treatment of a 46-year-old Saudi man with advanced stage liver metastatic neuroendocrine rectal cancer. The patient presented with a large liver lesion and rectal bleeding. He was cachectic, with a firm tender mass 20 mm above the anal verge. Computed tomography (CT) showed a mass 9.5 × 13 cm in size in the right hemi-liver, abutting the middle hepatic vein. The patient refused treatment, and consulted another hospital. After 3 months, he presented with the same symptoms in addition to delirium. Colonoscopy showed an ulcerating anorectal mass, from which a biopsy was taken. Repeat CT showed an increase in the size of the liver lesion to 17 cm and no change in the pelvis. The final histopathology report identified anaplastic small cell carcinoma. The patient underwent extended right liver resection followed by abdominoperineal resection, then 13 cycles of chemotherapy and monthly somatostatin injections. At the most recent follow-up, the patient had been disease-free for 48 months. Surgical resection (R0) of the primary and secondary tumor, followed by platinum-based chemotherapy can result in good survival in cases of small cell carcinoma with large liver metastasis, irrespective of whether the primary or secondary tumor is resected first.

## Background

Neuroendocrine tumors include a wide spectrum of lesions including microcarcinoid, carcinoid, and mixed endocrine/exocrine tumors, and small cell carcinoma (SCC). SCC most commonly originates in the lungs, but it can originate anywhere in the body, including the esophagus [1], large bowel [2] or urinary bladder [3], and is then referred to as extrapulmonary SCC.

Gastrointestinal (GI) SCC is a type of extrapulmonary carcinoma, and the GI tract is known to have the largest population of neuroendocrine cells [4]. Approximately 650 cases of SCC of the GI tract have been reported in the literature [5]. Colorectal SCC was reported to comprise 25% of all reported SCC cases of the GI tract [5], and the incidence of this carcinoma is less than 0.2% of all types of colorectal cancers [6].

Staging [7] and management [5] of GI tract SCC have been proposed and implemented in case reports and a few retrospective studies. The staging system was introduced by the Veteran’s Administrating Lung Study Group (VALSG) for primary SCC of the lung [8]. According to this system, SCC can be divided into two categories: 1) limited disease, in which the tumor is contained within a localized region; and 2) extensive disease, in which the tumor is outside the locoregional boundaries. The treatment for limited disease is local treatment in the form of radiotherapy, surgery, or both, plus platinum-based chemotherapy [7,9,10]. Distant metastasis (extensive disease) is treated with platinum-based combination chemotherapy [9,11].

Unfortunately, patients with SCC of the GI tract have a dismal prognosis, with a median survival of only 6 to 20 months [2,12,13]. Moreover, patients with liver metastasis have a median survival of a few weeks [14-16]. Thus, very few cases of colorectal SCC have been reported in the literature, Only three cases of colorectal SCC metastasizing to the liver have been reported and the outcome was extremely poor.

We therefore describe the first such case, to our knowledge, of successful surgical treatment of SCC of the rectum with advanced stage liver metastasis, followed by combination chemotherapy achieving long-term survival.

## Case presentation

A 46-year-old Saudi man was referred to Al-Hada Military Hospital, Taif, Saudi Arabia; he reported bleeding from the rectum for 6 months, along with decreased appetite, weight loss, and pain in the right upper quadrant of his abdomen. On examination, he was conscious and cachectic, with a mildly distended abdomen and a large hepatomegaly. Upon digital rectal examination, a firm and tender mass was found, 2 cm above the anal verge.

Laboratory tests including complete blood count, renal panel, liver function test, carcinoembryonic antigen, and α-feto-protein were all normal. A CT scan of the abdomen showed a huge mass (13.5 × 8 cm) in the right liver reaching down to the right iliac fossa and pushing the abdominal viscera away.

**Figure 1:**
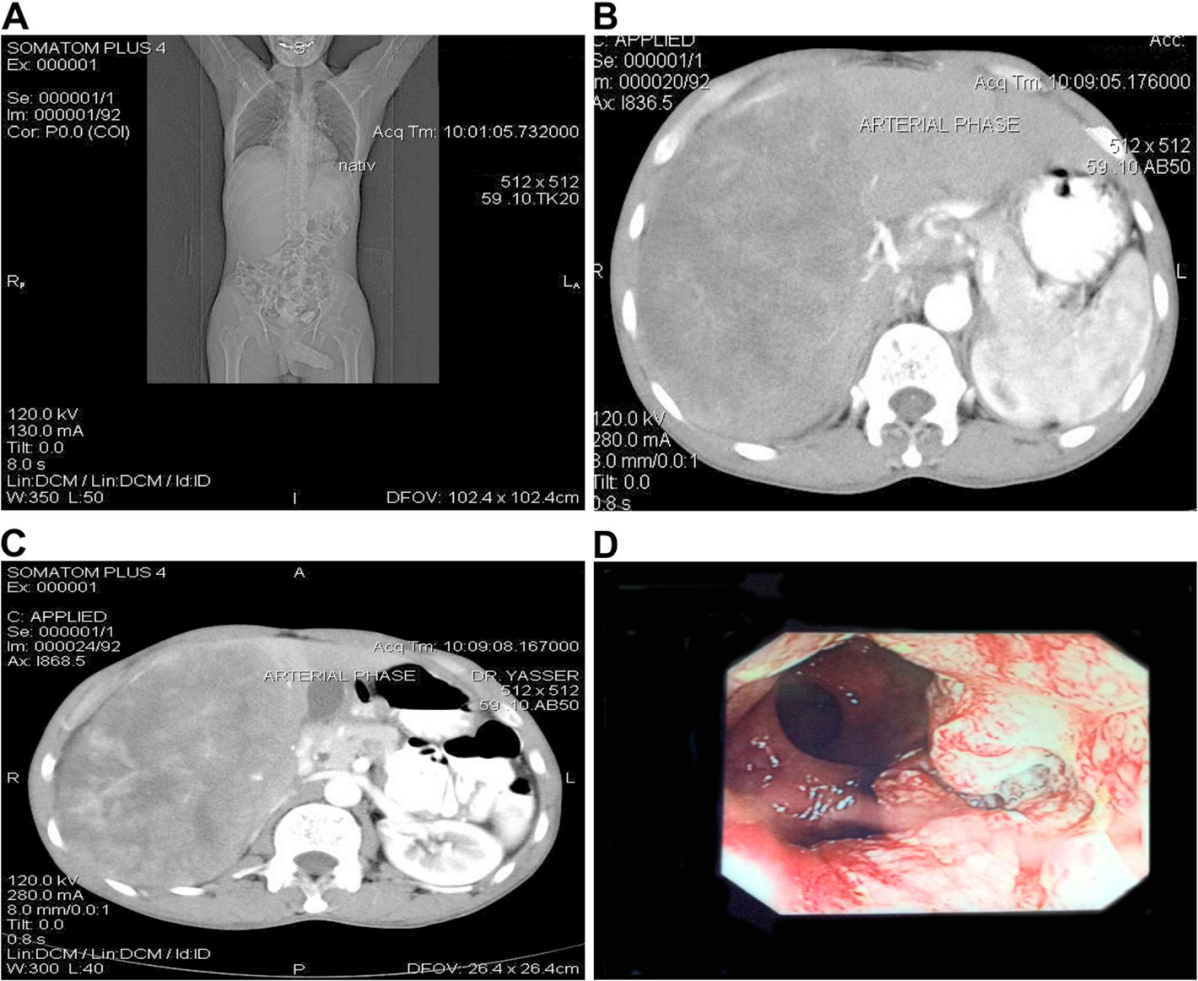
**Imaging of the liver and rectal masses.** (**A**) Scout film showing the large liver mass reaching down to the pelvis. (**B**) Computed tomography (CT) scan in the arterial phase showing the large liver mass involving the right hemi-liver, in close proximity to the main hepatic artery and involving the right hepatic artery. (**C**) CT scan in the venous phase showing the large liver mass compressing the right renal vein. (**D**) Colonoscopic image showing the mass lesion in the rectum.

Unfortunately, the patient refused to undergo colonoscopy, and was discharged against medical advice, because he wished to get a second opinion. Three months later, he returned to our hospital. He presented to the emergency department with delirium and impaired liver function (total bilirubin 47 g/dl (normal: 0 to 22 g/dl), alkaline phosphatase 203 μ/l (40 to 150 μ/l), aspartate aminotransferase 598 μ/l (5 to 35 μ/l), and alanine aminotransferase 373 (0 to 55 μ/l])). A repeat CT scan showed a dramatic increase in the size of the liver mass (Figure 1A), from 13.5 (maximum diameter) to 17 cm (Figure 1B,C). Colonoscopy showed an ulcerating mass 2 cm from the anal verge, extending up to 8 cm in length (Figure 1D). Biopsy examination of a specimen of the mass showed large, moulding pleomorphic nuclei and smooth hyperchromatic nuclear chromatin with a high rate of mitosis, suggestive of SCC of the rectum.

**Figure 2:**
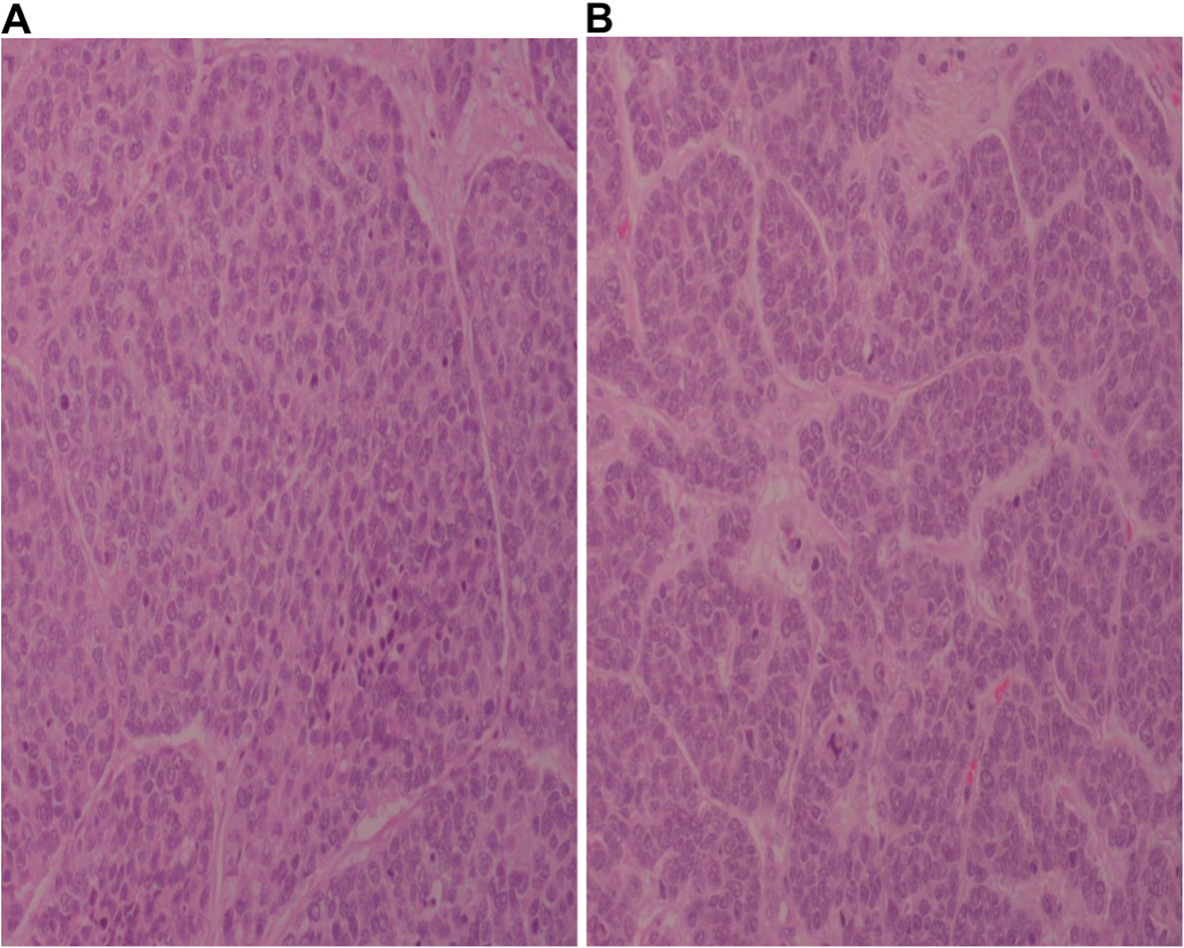
**Small cell carcinoma with large moulding pleomorphic nuclei and smooth hyperchromatic nuclear chromatin with a high mitosis rate.** Results from (**A**) rectum and (**B**) liver.

A multidisciplinary team came to a consensus on a two-stage procedure. In February 2009, the patient underwent extended right liver resection, which was followed by abdominoperineal resection in April 2009. On histopathological examination of the specimen, an SCC measuring 4 × 2 cm was seen arising at the anorectal junctio. The distal and proximal margins were tumor-free. The tumor was found to invade the entire thickness of the rectal wall (T4) (N0) (Figure 2A). Histopathological examination of the liver specimen (26 × 17 × 10 cm) showed it to be a poorly differentiated neuroendocrine carcinoma with clear margins (Figure 2B).

In August 2009, the patient received 13 monthly cycles of chemotherapy (cisplatin and etoposide), which was followed by monthly treatment with intrasmuscular somatostatin 20 mg, which is continuing. Positron emission tomography in May 2012 and CT in September 2012 did not show signs of recurrence. To date, the patient has been disease-free for 48 months.

## Discussion

Only a few cases of patients with limited rectal SCC with long-term survival have been published. While only three cases [14-16] with liver metastasis (Extensive disease) from which the patients died within weeks of the diagnosis, with or without treatment. Spiliopoulou *et al*. [7] reported a case in 2011 of a patient with an obstructing tumor in the lower rectum (limited disease) treated with a combination of chemotherapy and radiotherapy; this patient was disease-free 4.5 years after completion of the treatment. In 2004, Kim *et al*. reported a median overall survival of 15.3 months in a retrospective study of 24 patients [10]. In the same year, Brenner and colleagues [2] reported a series of 64 patients with GI tract SCC, and concluded that the disease had the highest incidence in the colon and rectum, had a tendency toward male predominance, and had a median survival rate of 6 to 12 months in treated patients and 6 to 12 weeks for untreated patients.

Only three cases of SCC of the rectum with liver metastasis have been published, and the patients were not treated surgically in any of these cases. In a report by Robidoux *et al*. [14], a patient with SCC of the rectum (limited disease) was treated with radiation and a multidrug regimen for SCC of the lung for 12 months. The patient then developed liver metastasis (extensive disease) and died within 2 months as a result of liver failure. Vilor *et al*. [15] reported a case of a highly aggressive neuroendocrine SCC of the rectum with liver metastasis (extensive disease); the patient died rapidly within 1 month from a widely extensive liver metastasis. The third case was reported by Anne in 2006 [16], in which a patient with locally advanced rectal SCC with liver metastasis (extensive disease) was treated with chemotherapy (cisplatin and etoposide) and palliative radiotherapy; however, the patient died 4 months after the diagnosis.

After reviewing the literature, we decided to attempt surgery on our patient because we could find no apparent significant advantages of chemotherapy or a combination of chemotherapy and radiotherapy in such cases (extensive disease), although this was based only on the three existing cases reported in the literature. Our treatment strategy was unique in that we started with the secondary tumor because the patient presented with deterioration of liver function, recent onset of delirium, and significant rapid growth of the liver with no change in the rectal tumor during the 3 months that elapsed between the patient deciding to seek another opinion and the time he re-presented to us. Surgery was followed by the established chemotherapy protocol (cisplatin and etoposide) to achieve the maximum result, followed by somatostatin therapy. This case is the first in which the patient had a huge liver metastasis of rectal SCC, and was successfully treated with surgery followed by chemotherapy. This treatment has so far resulted in long-term disease-free survival (48 months).

## Conclusion

Surgical resection (R0) of rectal SCC (limited or extensive disease), starting with the primary or secondary tumor, depending on the patient’s presentation, followed by (platinum-based) chemotherapy may result in better outcome and survival.

## Consent

Written informed consent was obtained from the patient for publication of this Case report and any accompanying images. A copy of the written consent is available for review by the Editor-in-Chief of this journal.

## Competing interests

The authors declare that they have no competing interests.

## Authors’ contributions

BOA-J was the primary treating surgeon, followed up the patient, and wrote the paper. OAM: collected all the data, helped in writing the paper, and followed up the patient in the ward during his treatment process. All authors read and approved the final manuscript.
